# Case Report: transcavernous sinus resection of parotid carcinoma with perineural invasion to Meckel’s cave and cavernous sinus

**DOI:** 10.3389/fonc.2026.1741177

**Published:** 2026-03-03

**Authors:** Li Cai, Ruben Dammers, Elise G. Rushing, Clemens M. F. Dirven, Ricardo Marian-Magaña, Ali F. Krisht

**Affiliations:** 1 Department of Neurological Surgery, Catholic Health Initiatives (CHI) St. Vincent North, Arkansas Neuroscience Institute, Sherwood, AR, United States; 2 Department of Neurosurgery, Center for Complex Microvascular Surgery, Erasmus MC Stroke Center, Erasmus University Medical Center, Rotterdam, Netherlands; 3 College of Medicine, University of Arkansas for Medical Sciences, Little Rock, AR, United States; 4 Department of Neurological Surgery, Hospital Civil de Guadalajara “Fray Antonio Alcalde”, Guadalajara, Mexico

**Keywords:** abducens nerve localization, adenoid cystic carcinoma (ACC), cavernous sinus surgery, internal carotid artery (ICA) skeletonization, Meckel’s cave, parotid gland carcinoma, perineural invasion (PNI), pretemporal transcavernous approach

## Abstract

**Background:**

Adenoid cystic carcinoma (ACC) of the salivary glands is a rare but aggressive malignancy known for perineural invasion (PNI), allowing tumor spread along cranial nerves to the skull base, Meckel’s cave, and cavernous sinus. Cavernous sinus involvement has traditionally been considered inoperable due to the density of neurovascular structures. Recent advancements in skull base microsurgery and anatomical landmark–guided navigation have enabled radical tumor resection in carefully selected patients.

**Objective:**

To describe a tailored pretemporal extradural transcavernous skull base approach, combined with infratemporal fossa exposure, for radical resection of parotid ACC with extensive PNI into Meckel’s cave and the cavernous sinus, emphasizing internal carotid artery (ICA) skeletonization, fascicular-level nerve dissection and repair, and precision bone work.

**Methods:**

A 54-year-old male presented with progressive left facial paralysis (House–Brackmann grade VI), trigeminal pain (V_1_), diplopia, and cranial nerve IV and VI palsies. MRI demonstrated a tumor extending along the mandibular nerve (V_3_) via the foramen ovale into Meckel’s cave and cavernous sinus, circumferentially encasing the cavernous ICA with patent lumen. High resolution neuroimaging confirmed perineural spread; PET-CT showed no metastasis. A modified Dolenc pretemporal extradural transcavernous approach was performed without orbitozygomatic osteotomy. Bone removal was limited to V_2_–V_3_ triangle drilling, exposing the infratemporal fossa and Meckel’s cave. ICA skeletonization was guided by the vidian canal, lingual petroclival ligament, and petrosphenoidal ligament, with intraoperative Doppler confirmation. Fascicular-level neurorrhaphy reconstructed infiltrated V_3_ fibers. Multilayer skull base reconstruction with autologous fat grafts provided watertight closure and radioprotection.

**Results:**

Gross total resection was achieved with preserved integrity of the ICA and all cranial nerves. No new neurological deficits occurred; preexisting palsies remained stable. Pathology confirmed ACC with extensive PNI; immunohistochemistry was positive for Hematoxylin and eosin, B-catenin, CK-7, and S100. Postoperative MRI verified complete cavernous sinus tumor removal. The patient recovered well and was referred for adjuvant stereotactic radiotherapy.

**Conclusion:**

This case demonstrates that even extensive cavernous sinus invasion by parotid ACC can be safely addressed with curative intent through precision-based microsurgery. A modified Dolenc approach with limited bone work and infratemporal extension enables maximal resection while preserving function. Strategic reconstruction optimizes safety for adjuvant radiation. This report adds to the growing evidence supporting aggressive yet function-preserving surgical management of malignant skull base tumors in multidisciplinary oncology programs.

## Introduction

Adenoid cystic carcinoma (ACC) of the salivary glands is a rare malignancy, representing approximately 1% of head and neck cancers and 10% of salivary gland tumors (1). Despite their slow growth, ACCs demonstrate highly invasive behavior with perineural invasion (PNI) occurring in more than 60% of cases (2, 3). PNI commonly extends along cranial nerves, particularly the trigeminal nerve, enabling the tumor to spread through the foramina ovale and rotundum to reach the skull base, Meckel’s cave, and the cavernous sinus (CS) (2–4). This pattern of extension is well documented and poses unique diagnostic and surgical challenges, often contributing to delayed diagnosis and high recurrence rates (1, 2).

Historically, CS involvement was considered a contraindication for resection because of its dense neurovascular anatomy, including cranial nerves III–VI and the cavernous segment of the internal carotid artery (ICA) (5, 6). However, significant advances in microsurgical skull base techniques, neuronavigation, and intraoperative vascular imaging have made aggressive but safe resection achievable in select cases (5, 6). The pretemporal extradural transcavernous (Dolenc) approach, refined by Krisht and colleagues, provides direct extradural access to the CS, enabling ICA skeletonization and precise cranial nerve dissection while minimizing morbidity (5, 6).

Previous clinical series have shown that radical surgical intervention can improve local control in ACC cases with intracranial extension. For example, Spetzler et al. reported gross or near total resection in four of five ACC cases with CS involvement using skull base approaches, including orbitozygomatic and infratemporal exposures (7). Gormley et al. similarly demonstrated that aggressive surgical management is feasible and can offer long-term disease control (8), while Ramakrishna et al. proposed an evidence-based multidisciplinary algorithm emphasizing surgical and adjuvant therapy integration (9).

Here, we present a rare case of parotid ACC with tumor spread along V_3_ into Meckel’s cave and the CS. We describe a modified Dolenc approach with infratemporal extension that achieved gross total resection without orbitozygomatic osteotomy, emphasizing anatomical landmark– guided ICA skeletonization, selective fascicular neurorrhaphy, and focused bone work. This case illustrates the evolution of surgical strategies for advanced skull base malignancies traditionally regarded as unresectable.

## Case presentation

A 52-year-old male was admitted with a six-month history of progressive left-sided facial paralysis, trigeminal pain in the V_1_ distribution, diplopia, and cranial nerve IV and VI palsies. Neurological examination revealed House–Brackmann grade VI left facial paralysis and severe pain in the ophthalmic (V_1_) distribution of the trigeminal nerve.

**Figure 1:**
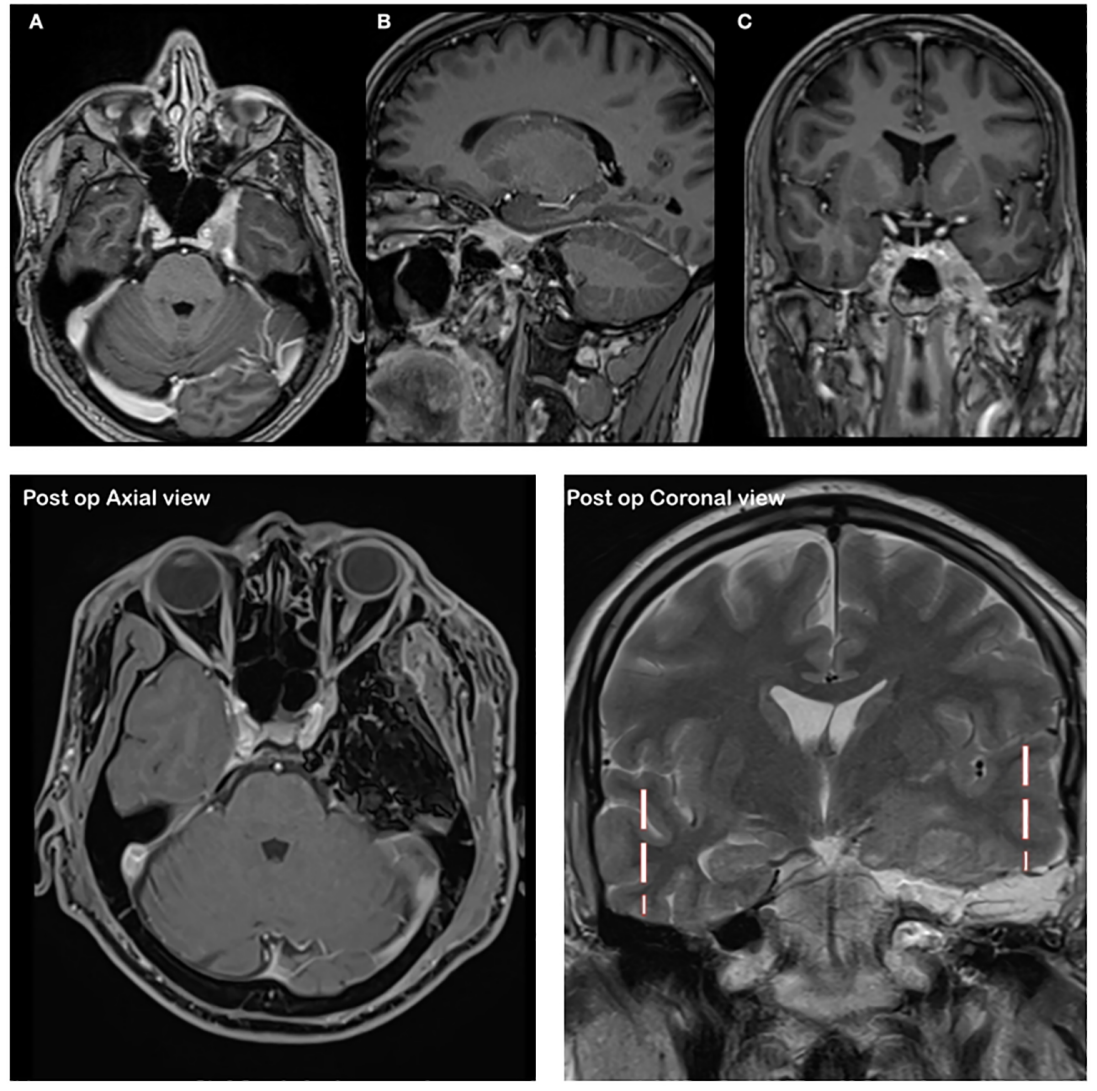
Preoperative axial **(a)**, sagittal **(b)** and coronal **(c)** T1-weighted post-gadolinium MRI demonstrating enhancing tumor extension along the mandibular division of the trigeminal nerve(V_3_) into Meckel’s cave and the cavernous sinus, with encasement of the internal carotid artery and evidence of perineural spread. Postoperative contrast-enhanced, fat-suppressed T1-weighted axial MRI demonstrating complete tumor resection with the surgical cavity filled by fat graft. Coronal MRI show natural elevation of the temporal lobe from the skull base, with excellent preservation of temporal lobe integrity.

Magnetic resonance imaging (MRI) demonstrated a mass extending along the mandibular division of the trigeminal nerve (V_3_), through the foramen ovale into Meckel’s cave and the cavernous sinus, and circumferentially encasing the cavernous segment of the internal carotid artery (ICA) while preserving lumen patency (**Figure 1**). The posterior tumor margin reached the petroclival junction without infratentorial involvement. High-resolution MR neurography confirmed cavernous sinus involvement with perineural spread along the trigeminal divisions, without evidence of clival bone destruction. Physical examination showed no evidence of cervical lymphadenopathy. A multidisciplinary skull base tumor board recommended radical microsurgical resection followed by adjuvant radiotherapy. The patient expressed significant relief regarding the resolution of preoperative trigeminal neuralgia and prioritized the preservation of masticatory function, which were achieved through the nerve-sparing approach.

## Surgical technique

A left pretemporal craniotomy was performed without orbitozygomatic osteotomy. The patient’s head was rotated > 45° to the contralateral side with slight extension, allowing the frontal and temporal lobes to fall naturally away from the skull base under gravity. A curvilinear preauricular incision was made to expose the zygomatic root, with meticulous preservation of the superior temporal artery and the frontal branch of the facial nerve. The temporalis muscle was sharply dissected in a subperiosteal plane under constant irrigation, mobilized, and reflected inferiorly as a thin, tension-free flap to maximize the operative corridor.

**Figure 2:**
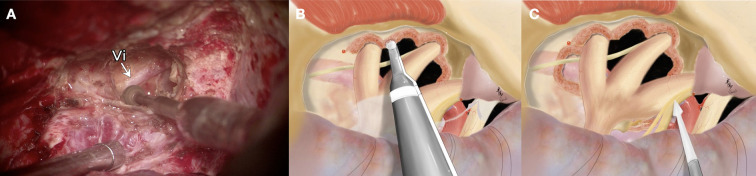
**(A)** Zoom-in surgical view demonstrating the pretemporal craniotomy and the targeted bone work strategy. The drilling is concentrated through the V2-V3 triangle, opening the foramen ovale to free the mandibular nerve (V3). This effectively transforms the middle cranial fossa floor from a structural barrier into a functional gateway to the infratemporal fossa. Vi=Vidian nerve. **(B, C)** Wide-angle view demonstrating detailed anatomical relationships showing the “downward extension” of the modified Dolenc approach. The exposure includes the zygomatic arch both anteriorly and posteriorly, with the temporalis muscle flattened and retracted inferiorly. This provides the necessary wide-angle access to ensure radical tumor clearance from the surrounding soft tissues and the trigeminal divisions, particularly the mobilized V3. These panels highlight the Vidian nerve (Vi) within the pterygoid canal as it relates to the sphenoidal sinus. The skeletonization of the internal carotid artery reveals the petrous-to-cavernous transition in relation to the petrolingual ligament.

A large pretemporal craniotomy was fashioned flush with the anterior skull base. Multiple burr holes were placed along the temporal squama, and the bone flap was elevated with care to preserve the dura. Wide extradural dissection exposed the entire middle cranial fossa floor. The V_2_–V_3_ triangle was extensively drilled to enlarge the foramina rotundum and ovale, establishing a direct extradural route to the infratemporal fossa and the tumor deposits tracking along V_3_. The lateral wall of the sphenoid sinus was opened for inspection, and bone removal posterior to the middle meningeal artery was minimized to maintain integrity of the temporomandibular joint (**Figure 2**).

The vidian canal and nerve served as constant anatomical landmarks for localizing the paraclival segment of the internal carotid artery (ICA), while the lingual, petroclival, and petrosphenoidal ligaments guided precise ICA skeletonization. Intraoperative Doppler ultrasonography confirmed ICA localization at the C5–C3 segments. The abducens nerve was identified and preserved in Dorello’s canal, and the oculomotor canal was fully unroofed to decompress CN III. Dual-layer tentorial dissection allowed safe mobilization of CN IV.

**Figure 3:**
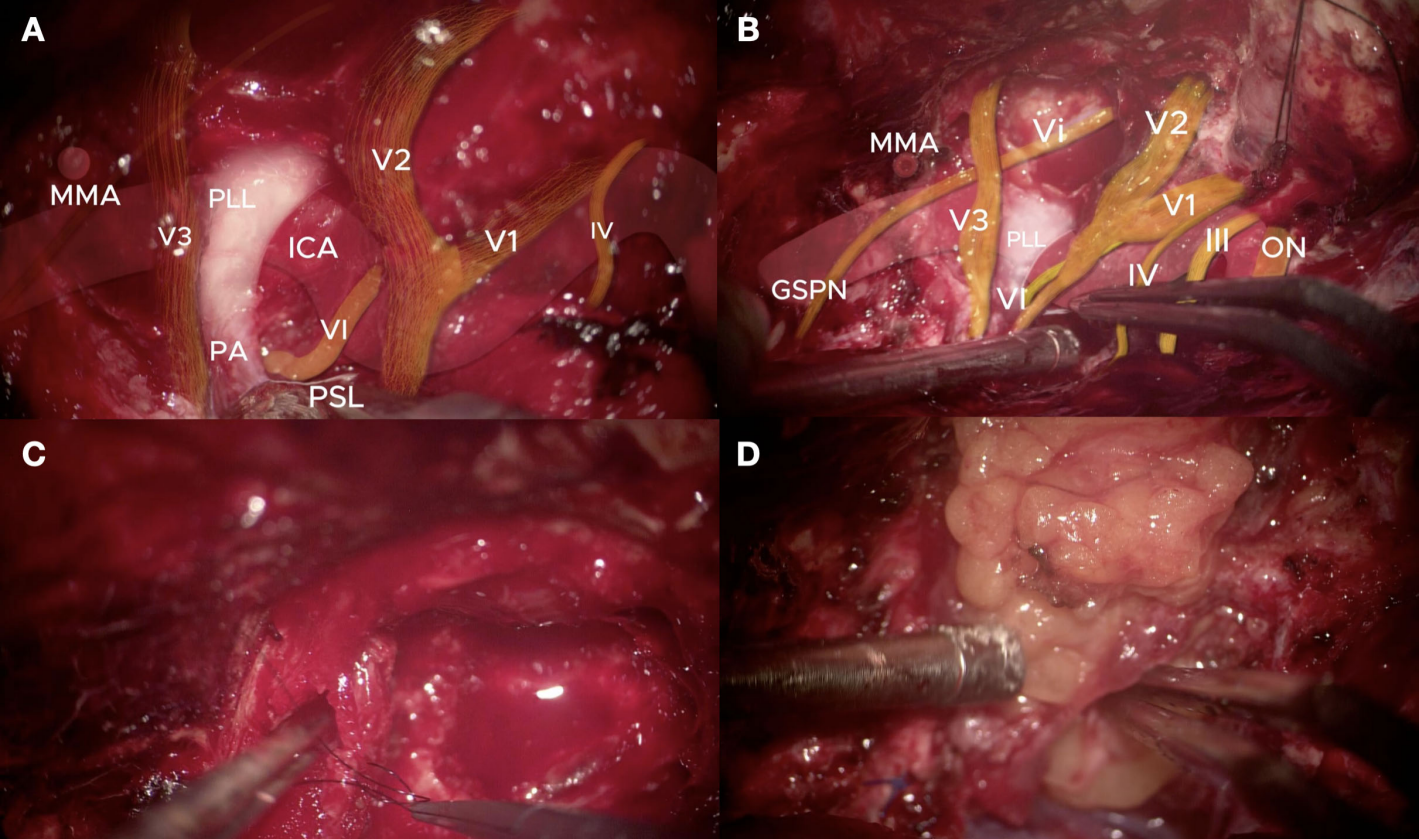
**(A, B)** Microsurgical anatomy of cavernous sinus, ICA skeletonization landmarks, abducens nerve in Dorello’s canal, and trochlear nerve mobilization. Vi=Vidian nerve; VI=Abducens nerve; ICA=Internal Carotid Artery; PA=Petrous Apex; PLL=Petrolingual ligament; PSL=Petrosphenoidal Ligament; MMA=Middle Meningeal Artery; GSPN=Greater Superficial Petrosal Nerve; ON=Optic Nerve; III=Oculomotor Nerve; IV=Trochlear Nerve; V1=Ophthalmic division of the Trigeminal Nerve; V2=Maxillary division of the Trigeminal Nerve; V3=Mandibular division of the Trigeminal Nerve. **(C)** Microsurgical repair of V3 nerve using 6–0 microsuture. **(D)** Fat graft used over the V3 nerve and for skull base reconstruction. Digital color overlays (yellow for the nerves and red for the internal carotid artery) were applied to the original microsurgical photographs to enhance the visualization of complex neurovascular relationships.

Tumor removal began with internal debulking, followed by meticulous sharp dissection along the ICA adventitia. The lesion was carefully peeled from CN III, IV, and V fascicles. Selective fascicular-level neurorrhaphy was performed for infiltrated V_3_ fibers to preserve continuity, extending from the trigeminal ganglion to the infratemporal fossa. Some V_3_ fascicles with direct tumor involvement were reconstructed with 6–0 monofilament microsutures. Tumor deposits in the posterior cavernous sinus compartments and Meckel’s cave were excised, and careful dissection between CN VI and the ICA ensured complete clearance (**Figure 3**).

Hemostasis was achieved using bipolar coagulation, fibrin sealant, and precise suturing of venous bleeding points along the cavernous sinus wall. Multilayer skull base reconstruction was performed with fascia lata duraplasty and autologous fat grafts interposed between critical neurovascular structures. These fat grafts provided radioprotection and watertight closure, reducing the risk of cerebrospinal fluid leakage and optimizing conditions for planned adjuvant stereotactic radiotherapy.

## Results

**Figure 4:**
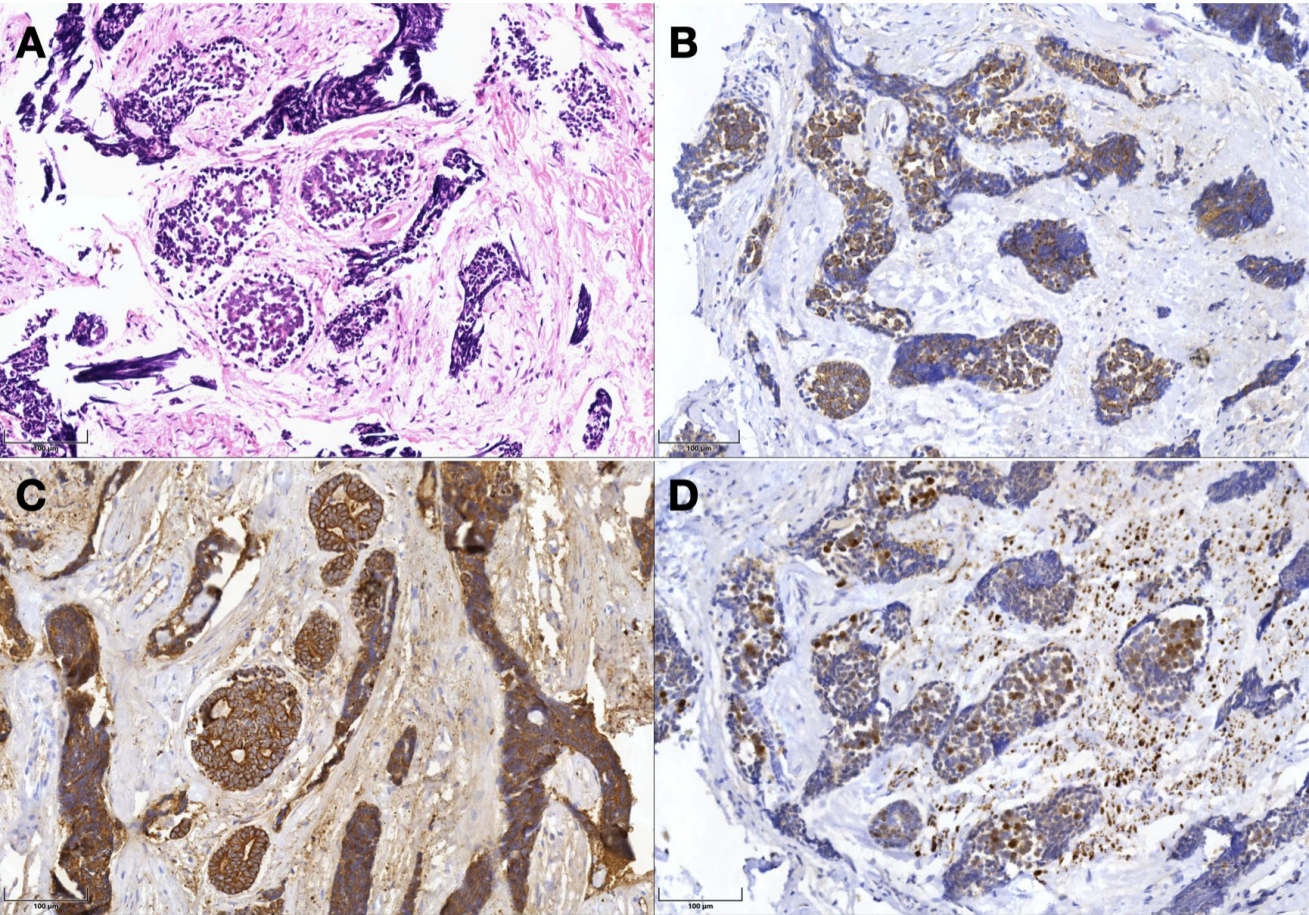
Histopathology with immunohistochemistry confirming ACC. **(A)** Hematoxylin and eosin. **(B)** B-catenin. **(C)** CK-7. **(D)** S.100.

Postoperative MRI confirmed gross total resection of the tumor, with complete clearance of Meckel’s cave and cavernous sinus and preserved ICA lumen (**Figure 1**). There were no new cranial nerve deficits; preexisting facial paralysis and diplopia remained unchanged. MRI enhanced and histopathology revealed classic cribriform-pattern adenoid cystic carcinoma with prominent perineural invasion, and immunohistochemistry was positive for Hematoxylin and eosin, B-catenin, CK-7, and S100, consistent with ACC (**Figure 4**).

The patient was discharged home on postoperative day 3, with intact wound healing and stable neurological status. Multidisciplinary follow-up recommended adjuvant radiation therapy targeting the parapharyngeal space and skull base with image-guided planning, aided by intraoperative fat graft placement. At the 6-month postoperative evaluation, the patient required a tarsorrhaphy performed by the ophthalmology service to protect the cornea, as peripheral facial nerve palsy persisted. Despite this sequela, the overall clinical course has been favorable. Careful examination demonstrated that ocular motility remained intact in both eyes, and movements of the left globe could still be appreciated through the eyelid despite the tarsorrhaphy. This finding strongly suggested preserved function of cranial nerves III, IV, and VI, highlighting the successful maintenance of extraocular motor function despite the extent of the tumor resection.

The patient denied trigeminal neuralgic pain in the ophthalmic (V1), maxillary (V2), and mandibular (V3) distributions, which is noteworthy given the proximity of the surgical field to the trigeminal nerve. Nevertheless, he reported residual hypoesthesia and numbness limited to the mandibular (V3) territory, consistent with partial sensory involvement rather than painful neuropathy. Overall, his postoperative condition illustrates a favorable neurological outcome, with preserved ocular motor function, absence of disabling pain, and limited sensory deficit as the main residual symptom.

## Discussion

ACC of the salivary glands is a rare malignancy characterized by deceptively slow growth yet highly invasive biological behavior, most notably marked by PNI (10, 11). PNI facilitates tumor spread along cranial nerves, most commonly the trigeminal nerve, allowing intracranial extension to Meckel’s cave and the CS through the foramina ovale and rotundum (12, 13). This pattern of infiltration poses significant complications to surgical management, as tumors frequently encase the cavernous segment of the ICA and involve cranial nerves III–VI. These features are historically regarded as contraindications to resection (14).

Advancements in microsurgical skull base techniques, combined with high-resolution MRI, anatomical neuronavigation, and intraoperative Doppler ultrasonography, have redefined the management of such complex lesions (15, 16). Dolenc’s seminal description of the pretemporal extradural transcavernous approach established a direct extradural route to the CS, permitting safe ICA skeletonization and precise cranial nerve dissection. Subsequent refinements by Krisht and colleagues emphasized early extradural dissection, selective bone removal, and cranial nerve mobilization, further expanding the applicability of this approach for both vascular and oncologic surgery (17).

In the present case, a modified Dolenc extradural transcavernous approach was combined with infratemporal fossa exposure to address extensive perineural spread from the parotid gland, along the V3 nerve, into both the CS and infratemporal compartments. Gross total resection was achieved, including removal of tumor tissue circumferentially encasing the cavernous ICA, while preserving the abducens nerve and other critical neurovascular structures. This microsurgical strategy prioritized safe manipulation of the ICA, stepwise dissection of the cranial nerves, and precise orientation of the anatomy to maximize tumor clearance without increasing morbidity.

While the endoscopic endonasal approach has revolutionized the surgical management of the medial cavernous sinus, its primary limitation in treating ACC is the restricted lateral “line-of-sight.” ACC infiltration does not respect midline boundaries; rather, it characteristically migrates laterally along the branches of the trigeminal nerve. The modified Dolenc approach was selected because it treats the middle cranial fossa floor as a gateway rather than a barrier. By concentrating bone removal on the V2-V3 triangle, the surgeon achieves simultaneous proximal and distal control of the internal carotid artery (exposure of both petrous and cavernous segments) and maintains neural continuity, allowing for the tracking of V3 from the extracranial infratemporal fossa(ITF) directly into Meckel’s cave. Standard transcavernous approaches typically terminate at the foramen ovale. However, in this case, addressing the extension into the ITF was paramount. By integrating the Dolenc corridor with an infratemporal/preauricular exposure, the surgical team avoided blindly “tugging” the nerve through the bony ostia. Instead, the tumor was sharply dissected away from the surrounding pterygoid muscles and the ICA, ensuring radical clearance at the critical transition point where the tumor moves from the extracranial to the intracranial space.

Several key technical principles supported the success of this surgery. First, anatomical landmark–based orientation was fundamental: the petrolingual ligament (PLL) and petrosphenoidal ligament served as highly reliable extradural markers for ICA localization (18, 19). Second, dual-layer tentorial dissection allowed early visualization and safe mobilization of the trochlear nerve, while decompression of the oculomotor canal alleviated nerve compression from tumor deposits (20). Third, selective fascicular dissection with end-to-end neurorrhaphy was used to preserve functionally intact mandibular nerve (V3) fibers while excising infiltrated fascicles, maintaining mastication function postoperatively (21). Fourth, in this case, despite the tumor circumferentially encasing the cavernous ICA, the abducens nerve (VI) was preserved through meticulous microsurgical dissection. Unlike the oculomotor and trochlear nerves, which reside within the lateral wall of the cavernous sinus, the abducens nerve is situated directly within the sinus, immediately adjacent to the ICA. This unique anatomical position, combined with its anchoring at Dorello’s canal, leaves the nerve with no “buffer space” to retreat from mass lesions, making it highly vulnerable to mechanical compression or tumor infiltration (22). By identifying the lateral trajectory of the ICA early via the petrolingual ligament (PLL) and petrosphenoidal ligament, we achieved precise localization and decompression of this fragile structure.

Bone removal was intentionally minimized to reduce approach-related morbidity: rather than performing an extensive orbitozygomatic osteotomy or petrous apex drilling, a focused extradural corridor was utilized by drilling the V_2_–V_3_ triangle and enlarging both foramina rotundum and ovale. This limited bone work preserved structural integrity, shortened operative time, and maintained optimal working angles for safe ICA skeletonization and tumor dissection. This approach reflects the philosophy of modern skull base surgery, favoring anatomical precision and minimally disruptive exposure over traditional wide osteotomies.

Reconstructive and oncologic planning were integrated preoperatively. Given ACC’s well-established propensity for recurrence, adjuvant radiotherapy was considered a critical component of disease control (14, 15). Autologous fat grafts were strategically positioned between preserved cranial nerves and high-risk tumor beds, creating a durable biological barrier to shield neurovascular structures during postoperative radiation (20). This strategy allowed high-dose stereotactic radiotherapy to be safely administered with minimized risk of radiation-induced neuropathy.

Overall, this case illustrates that in experienced skull base centers, CS involvement in ACCs should no longer be viewed as a contraindication to surgery (13, 16). Instead, these cases should urge utilizing advanced microsurgical strategies, precision anatomical dissection, and proactive radiation planning; all techniques shown to achieve radical tumor clearance with preservation of neurological function in select cases. The detailed technical nuances reported here—including nerve-sparing dissection, targeted bone removal, and reconstructive measures—provide valuable insight into the evolving paradigm for the management of aggressive skull base malignancies.

## Limitations

Although gross total resection was achieved in this case, several limitations persist: First, as a single case report, the long-term clinical benefits of this modified approach necessitate validation through larger-scale clinical studies. Second, the procedure demands an exceptionally high level of microsurgical skull base expertise, presenting significant challenges regarding technical reproducibility and the extensive training time required for proficiency.

## Conclusion

This case demonstrates the safe and effective application of a modified Dolenc extradural transcavernous approach with infratemporal extension for radical resection of parotid ACC with extensive PNI to Meckel’s cave and the cavernous sinus. Gross total resection was achieved while preserving cranial nerve function and ICA integrity through landmark-guided dissection, selective neurorrhaphy, dual-layer tentorial separation, and targeted bone work limited to the V_2_–V_3_ triangle. Autologous fat grafting provided radioprotection and enabled safe adjuvant therapy. These techniques demonstrate that even advanced CS involvement can be managed with curative intent using precision, nerve-sparing skull base surgery.

## Data Availability

The original contributions presented in the study are included in the article/Supplementary Material. Further inquiries can be directed to the corresponding authors.
